# Supplementary material for: Generation of Human Regulatory Dendritic Cells from Cryopreserved Healthy Donor Cells and Hematopoietic Stem Cell Transplant Recipients
Source: Cells. 2023 Sep 28;12(19):2372. doi: 10.3390/cells12192372 (PMC10571850; doi:10.3390/cells12192372)
Supplement: Supplementary file 1 [file cells-12-02372-s001.zip › cells-2581153-supplementary.pdf]

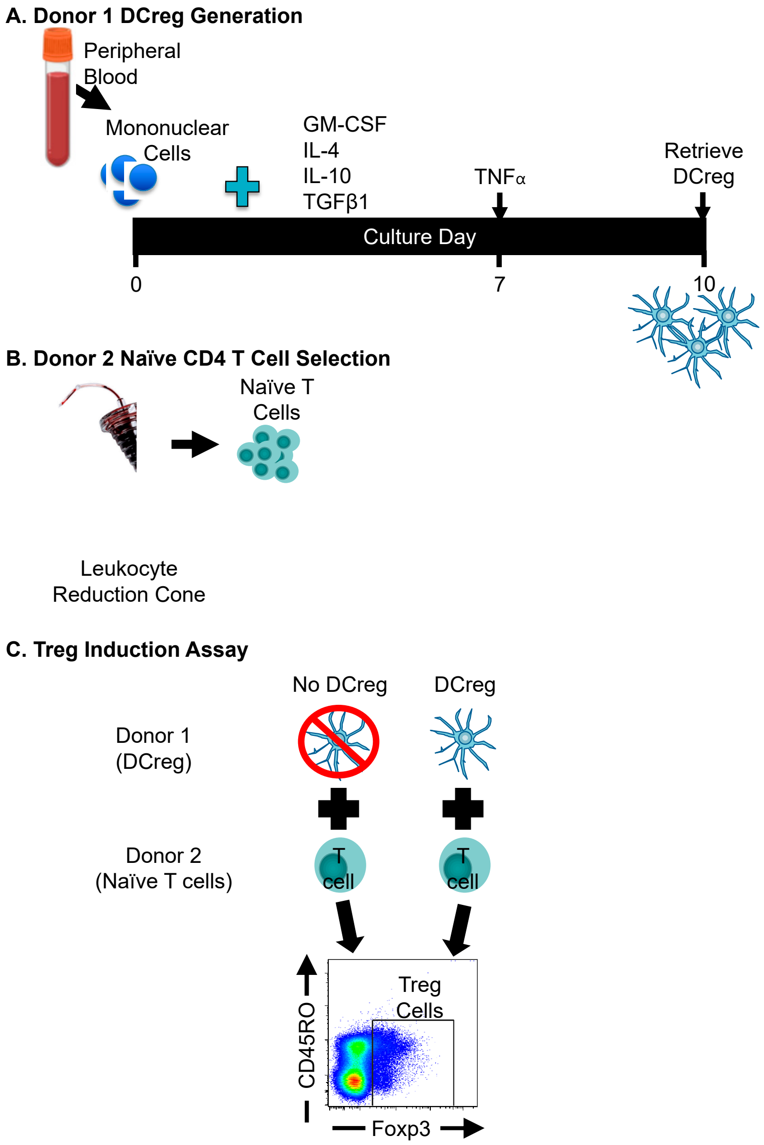

Supplementary Figure S1. *In Vitro* Treg induction Assay. (A) Generation of human DCreg from peripheral blood of donor 1. (B) Naïve donor CD4+ T cells for the Treg induction assay are negatively selected from leukocyte reduction system cone of donor 2. (C) Donor 2 CD4+ T cells are co-cultured with or without Donor 1 DCreg. After 5 days in co-culture, cells are isolated from the culture and stained for CD45RO and Foxp3. The frequency of Treg is determined via Foxp3+ expression.

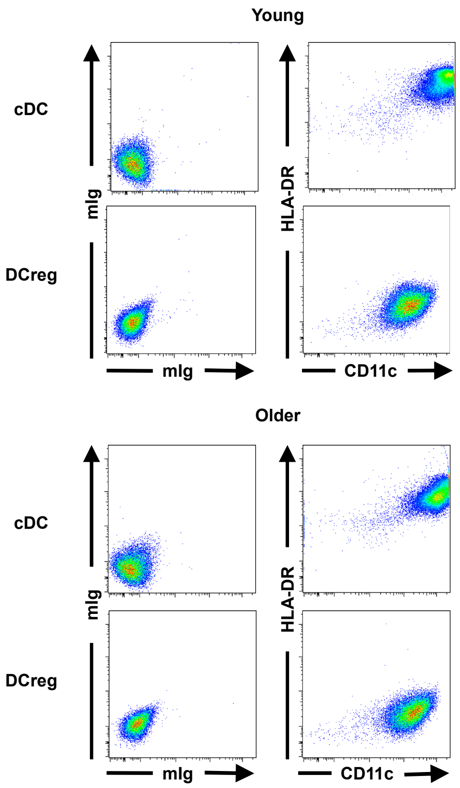

Supplementary Figure S2. Compared to cDC, young and older human DCreg generated from healthy donors display reduced expression of HLA-DR and CD11c. mIg = mouse isotype control antibody. Representative plots are shown. N ≥ 8 donors per age group.

56 Total Patients		
**Age**	**Number of Patients**	**Disease State**
<30	12	ALL (7), AML (2), CML (1), NHL (1), MDS (1)
≥50	44	ALL (7), AML (12), CLL (3), CML (3), MDS (12), Myelofibrosis (1), NHL (6)

Supplementary Table S1. Total number and hematological disease of young and older patients.

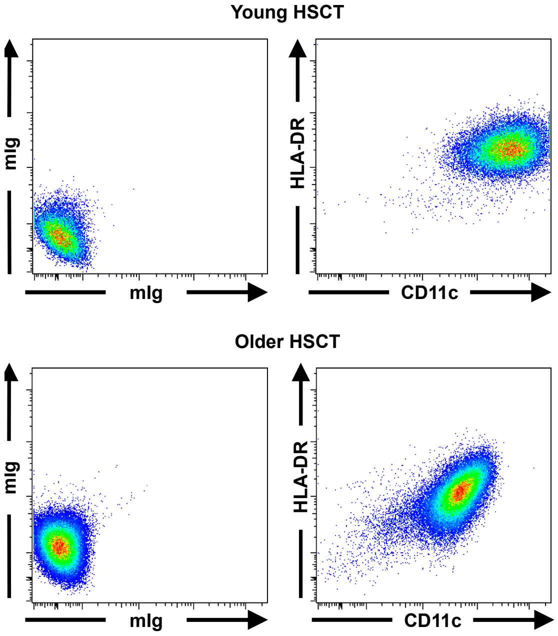

Supplemental Figure S3. Young and older HSCT patient samples generate DCreg that expressHLA-DR and CD11c at levels similar to healthy young and older DCreg. DCreg were generated from young and older monocytes isolated from the peripheral blood of patients upon admission to the hospital for HSCT. mIg = mouse isotype control. Representative plots are shown. Young N = 10; Older N = 23.

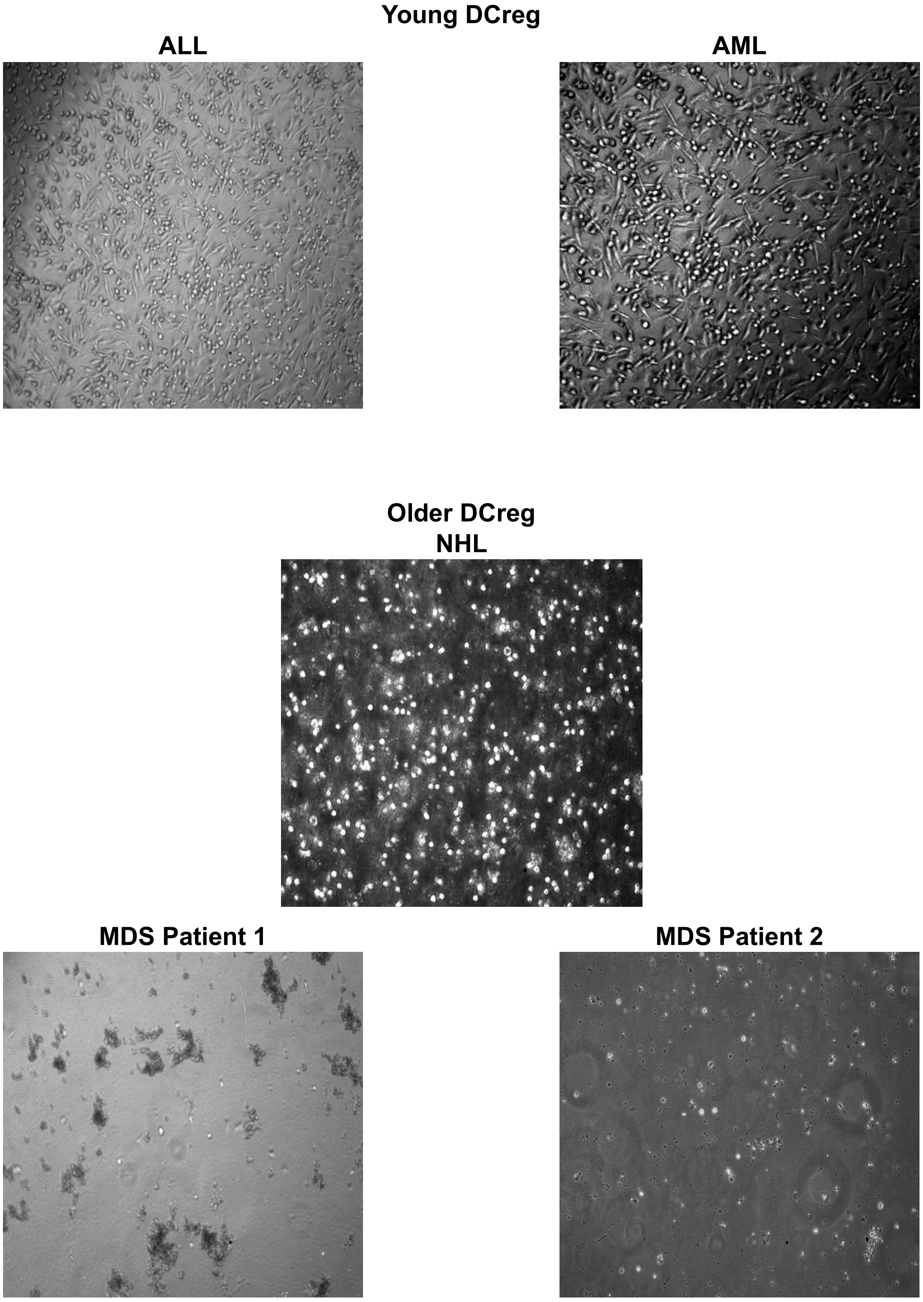

Supplemental Figure S4. Type of hematological disease impacts the generation of DCreg from HSCT patients. DCreg generated from monocytes purified from ALL, AML, and NHL patients showed round and elongated spindle shaped cells, while DCreg generated from MDS patients were irregular in shape with large masses of debri. Live cell images (40X) using a phase contrast inverted microscope were taken at the end of culture. MDS images are representative of MDS patients. The ALL, AML, and NHL images are representative of the ALL, AML, CML, and NHL (non-MDS) patients in the study. Young non-MDS N = 11. Young MDS N = 1. Older non-MDS N = 24. Older MDS N = 7.
